# Evidence-based ethics – What it should be and what it shouldn't

**DOI:** 10.1186/1472-6939-9-16

**Published:** 2008-10-20

**Authors:** Daniel Strech

**Affiliations:** 1 Institute of Medical Ethics, University of Tübingen, Schleichstraße 8, 72076 Tübingen, Germany

## Abstract

**Background:**

The concept of evidence-based medicine has strongly influenced the appraisal and application of empirical information in health care decision-making. One principal characteristic of this concept is the distinction between "evidence" in the sense of high-quality empirical information on the one hand and rather low-quality empirical information on the other hand. In the last 5 to 10 years an increasing number of articles published in international journals have made use of the term "evidence-based ethics", making a systematic analysis and explication of the term and its applicability in ethics important.

**Discussion:**

In this article four descriptive and two normative characteristics of the general concept "evidence-based" are presented and explained systematically. These characteristics are to then serve as a framework for assessing the methodological and practical challenges of evidence-based ethics as a developing methodology. The superiority of evidence in contrast to other empirical information has several normative implications such as the legitimization of decisions in medicine and ethics. This implicit normativity poses ethical concerns if there is no formal consent on which sort of empirical information deserves the label "evidence" and which does not. In empirical ethics, which relies primarily on interview research and other methods from the social sciences, we still lack gold standards for assessing the quality of study designs and appraising their findings.

**Conclusion:**

The use of the term "evidence-based ethics" should be discouraged, unless there is enough consensus on how to differentiate between high- and low-quality information produced by empirical ethics. In the meantime, whenever empirical information plays a role, the process of ethical decision-making should make use of systematic reviews of empirical studies that involve a critical appraisal and comparative discussion of data.

## Background

The concept of "evidence-based ethics", modeled after the concept of evidence-based medicine, [1] has increasingly found application in international journals in the past decade, ranging from a relatively uncritical use of the term [2-5] to attempts at its explication [6,7] to variously justified repudiations of the term [8,9]. However, so far this discussion has been lacking a thorough explication of the term "evidence-based" (EB) and the concept behind it. EB means more than one might suspect from a translation one meets with frequently, roughly speaking: "based on the latest and best available empirical information". For example, we see a relatively trivial definition of evidence-based ethics along these lines in Pascal Borry et al.: "Ethical decision making must necessarily be based on the use of the latest and best available medical research findings" [6]. Alongside this relatively unspecific explication of the concept of EB, the discussion so far also lacks an analysis of the practical problems that threaten to arise on any non-trivial determination of what evidence-based ethics might mean.

The concept of EB was first used in 1992 in the context of (clinical) medicine [10]. In the following years the term was increasingly extended to other areas far removed from the medical clinic. The most frequently cited characterization of evidence-based medicine (EBM) comes to us from David Sackett and was published in 1996 in the British Medical Journal (BMJ) under the title "Evidence-based medicine. What it is and what it isn't". However, the article failed to do justice to the various normative dimensions inherent in the EB concept, and as of today these have hardly been explicitly discussed and analyzed in any conceptual work on EBM. This implicit normativity holds not just for medicine (EBM) but equally for all those areas of study that have already been enriched by the EB concept or might be in the future. With the arrival of the EB concept in medical ethics or bioethics at the very latest our specialized discussion should explicitly identify these normative aspects and subject them to critical analysis. To guarantee the responsible employment of a reasonable and non-trivial reading of the concept, the following will expand on Sackett's descriptive characterization of EBM ("what it is and what it isn't") and discuss what an evidence-based ethics "should and shouldn't be". In contrast to the non-trivial interpretation that follows, a trivial reading would be a definition of evidence-based ethics that amounts to simply taking empirical information into account in ethical decision-making without specifying this any further (see above).

To provide a more accurate picture of the challenges and peculiarities of evidence-based ethics, this article is divided into three sections. The first is a systematic presentation of central descriptive and normative dimensions inherent in a non-trivial reading of the EB concept. The second section will then discuss the ethical problem areas associated with these normative dimensions using examples from EBM. In a final step these practical problems will be mapped onto the particularities of applied ethics (such as the concept of evidence in interview or other socio-empirical research) in order to clarify the challenges and limits of evidence-based ethics.

The critical analysis of the particularities of evidence-based ethics is important to ward off the potential misuse of the EB concept in medical ethics in a timely manner. But beyond this the results of this analysis are also significant for two further debates within modern bioethics. On the one hand, the results help to clarify more precisely how ethics conceives itself in its relation to empirical data per se [11]. And on the other hand, they will shed light on the relevance of ethical and methodological problems in assessing the quality of empirical ethics research in practice [12,13].

## Discussion

### Descriptive dimensions of "evidence-based"

In Sackett's definition, EBM is described in a more general version as follows:

"Evidence-based medicine is the conscientious, explicit and judicious use of current best evidence in making decisions about the medical care of individual patients. The practice of EBM means integrating individual clinical expertise with the best available external clinical evidence from systematic research." [1] p. 71

**Figure 1:**
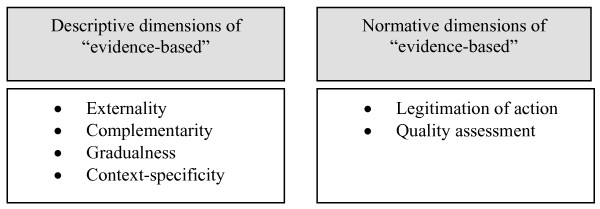
Descriptive and normative dimensions of the concept "evidence-based".

In the context of evidence-based ethics it is not the specification of EB for medicine that interests us so much but the characterization of what evidence or EB means in general. Textbooks and other writings on EBM provide us with further specifications of the content and the scope of the EB concept [14]. The following derives several characteristic descriptive dimensions of the concept from these specifications, and supplements them with the normative dimensions not explicitly discussed by Sackett and other authors (see figure 1).

#### Externality

In medicine and medical ethics we encounter empirical information in various forms, such as quantitative and qualitative indications of the benefit and harm of medical measures, reports of the number of organ donations, or on the level of satisfaction with clinical ethics consultation. This empirical information can be divided into the information that one has garnered in the course of one's life and professional experience (thus internally) and information that others have garnered (externally) in studies. As emphasized in the quotation from Sackett, the concept of evidence in EB stands for externally generated empirical information.

#### Complementarity

External evidence alone cannot influence any decisions – it always exists in a complementary relation to context-specific framing conditions, ethical principles and other decision-theoretical elements. Furthermore, typically various pieces of empirical information play a role in decisions. In the medical context, besides the internal and external information on the benefit of a measure, the individual or group-specific preferences of the patients in particular should also be taken into consideration. Complementary elements in ethical decisions also include context-sensitive ethical principles or norms (see below).

#### Gradualness and context-specificity

On a reasonable, non-trivial reading evidence (which is external) cannot be simply equated with empirical information per se. Not all available empirical information on a certain question counts directly as evidence according to the EB concept. Empirical information has to hold up under critical appraisal, a sort of qualifying exam, in order to be accepted into the higher class of evidence. Sackett writes:

"Because the randomised trial, and especially the systematic review of several randomised trials, is so much more likely to inform us and so much less likely to mislead us, it has become the 'gold standard' for judging whether a treatment does more good than harm. [...]And if no randomised trial has been carried out for our patient's predicament, we must follow the trail to the next best external evidence and work from there." [1] p. 72

According to this conception of EB only the available information that is sufficiently reliable (as determined by context) and demonstrates internal and external validity can be called evidence. This point is of central importance – in principle each question prompts a new decision about which information is to be considered evidence. The reliability determines how exactly a study measures a certain characteristic. There are various measures for quantifying the reliability of a test that cannot be individually discussed here [15]. The criterion of validity is divided into internal and external validity. Internal validity pertains to the credibility of the results within the study. In view of the diverse possible sources of systematic bias and the influence of chance, the EB concept demands that we only consider the results of those studies that reduce the risk of systematic bias and the influence of chance as much as possible. The external validity, on the other hand, describes the validity of the results outside the population considered by the study. Thus external validity is often used as a synonym for generalizability.

Hence there is a gradual relationship between empirical information per se and the empirical information that the EB concept considers evidence. In the field of medicine, for example, this often leads to a situation where, despite the availability of results from studies on the benefit of a certain medical procedure, its efficacy or effectiveness is not considered evidence-based, since the quality of the studies (their internal validity) or the generalizability of their results (external validity) have not been judged satisfactory. Here we have to keep in mind that medical and statistical experts often arrive at different answers to the central question: is the effectiveness of this specific medical intervention evidence-based or not? Examples of this include the controversy surrounding the early detection of breast cancer with mammography screening [16] or medication for the treatment of Alzheimer's disease (cholinesterase inhibitors) [17]. How to best approach this situation of a dispute among experts is at present an unresolved problem in EBM and medical ethics that has generated astonishingly little discussion [18]. This last problem in particular is closely related with the normative dimensions of the EB concept discussed below.

### Normative dimensions of "evidence-based"

#### Legitimation of action

The EB concept includes the normative dimension of the legitimation of action. Whenever empirical information plays a role in decision-making (be it medical, ethical, or health policy decisions), according to the EB concept the information should be given preference that fits or best fits the criteria of evidence (see above). Empirical information that reaches the highest EB status should be trusted more in action-oriented decisions than empirical information that does not satisfy the criteria of evidence. The legitimation of action that this involves does not arise on its own, nor are we dealing with a naturalistic fallacy. Rather the typical case is that previous specification of principles [19] and other deliberative processes determined what empirical information is needed to arrive at a rational decision through ethical principles. Thus in medical and ethical decision-making, for example, the benefit of a medical procedure plays a central role. Very often it is not clear whether a procedure produces significant and clinically relevant benefits, despite the availability of studies. However, should it be determined at a later time with newer and better studies that the benefit of the procedure is evidence-based, this will count strongly in favor of legitimating the use of the procedure as well as its funding by health insurance. This situation would only present us with a naturalistic fallacy if the significance of medical benefit for the decision-making process were not determined in advance and thus was already posited as a normative judgment.

#### Quality assessment

The decision as to what should be considered evidence is based on the quality of the underlying studies, i.e. their reliability and validity. Only the empirical information supported by a context-sensitive study of appropriate quality is to be considered evidence. But who can or should decide, using which criteria, whether the quality standards have been met or not? In actual medical practice and in empirical ethics we will have to continually lower the bar, since the perfect study without any susceptibility for systematic bias does not exist. This holds for experimental studies that generate the greater share of evidence in medicine and even more for qualitative studies and survey research that, according to the overview taken by Borry et al., represent the most common form of empirical research in ethics [20]. In many cases a high internal validity and a high external validity are mutually exclusive. So where should the optimal quality of study be pegged at? How far can the optimal quality be removed from the maximum? These decisions in assessing the quality of empirical information and their underlying studies imply various value judgments concerning the relevance of outcome parameters to the patient, the weighing of costs and benefits, the tolerance of uncertainty due to suboptimal study quality, and others [18]. With regard to the tolerance of uncertainty, for instance, we must acknowledge that every choice in this regard requires balancing the uncertainty of being wrong in our inferences about study quality with the probability of missing important information from studies of suboptimal quality. The answer to how much uncertainty in study quality we are willing to accept ought to be dependent on the context (e.g., severity of disease, existence of alternatives) and on the preferences and values of the particular stakeholder population to which the empirical information will be applied. Because there is no "one size fits all" approach for determining how much uncertainty should be tolerated in designing clinical studies or survey research, it becomes important for users of empirical data to be given more information about the investigators tolerance of uncertainty and their rationale for their choices in a given circumstance. A more thorough analysis of these various value judgments is beyond the scope of this paper. For a systematic analysis of these value judgments, see [18,21].

### Evidence and consensus

In the following the descriptive and normative dimensions of the EB concept will serve as a framework for a critical analysis of the associated challenges in general and for an evidence-based ethics in particular. It is important to keep in mind that the basic idea underlying the EB concept deserves our strong endorsement from an ethical perspective. When empirical information plays a role in decision-making, it should be weighted differently depending on its quality. However, from an ethical perspective the application of the EB concept becomes problematic when it is used uncritically or misused in order to legitimate actions. When a group of experts determines that the benefit of a certain medical procedure is evidence-based, this has a strong legitimating effect on certain actions at present. Doctors could come into conflict with their liability in civil law if they do not take evidence-based action, and insurance companies find it much harder to justify themselves if they wish to not fund evidence-based procedures. Yet before we can determine which empirical information deserves the "evidence-based" seal of quality, we first need context-specific standards for the optimal or at least sufficient internal and external validity. The search for consensus on such standards runs into significant problems in the case of internal validity, and the question of external validity only exacerbates them. Within EBM, for example, it is a matter of contentious debate whether the demands for internal validity are met by the use of a certain experimental study design (randomized controlled trials) or whether further aspects have to be considered (e.g. the dropout rate of the study participants) [22]. With the qualitative and quantitative empirical studies in medical ethics we can expect analogous controversies concerning the optimal methods of sampling, the evaluation of survey questions or the best ways to carry out interviews and analyze and interpret the results [23].

Depending on how strict we make the criteria for the needed empirical information to count as evidence-based, we will come to different conclusions about the underlying question (In EBM the question at issue is generally the quantitative and qualitative extent of benefit and harm from certain medical interventions. In empirical ethics (mostly interview research) the question is to determine majority views or to analyze opinions and attitudes). The selection and concretization of these criteria always involves normative judgments (see above). This would not in itself be so problematic, were it not for the fact that in practice we see various decision-makers frequently working with different criteria of assessment – hence the need to make these normative judgments transparent [18]. If they are not made sufficiently transparent, as we unfortunately see most of the time in practice, there is an ethically problematic latitude in the EB concept that allows for manipulation, namely: the exploitation of the EB concept for the dimension of *legitimation of action *even though there is no sufficient consensus regarding the relevant normative values posited in the *quality assessment *dimension. In other words: decisions can then be legitimated under the "evidence-based" seal of approval even though there is no consensus concerning what should count as evidence and what not in the particular context. Of course, this also works the other way round: The EB concept can also be exploited to argue against certain actions, e.g. the clinical use or coverage of medical interventions.

These considerations should have made it clear that the often criticized dimension of legitimation of action is not, in itself, the real ethical problem with the EB concept. Quite the contrary, it represents the reasonable and yet crucial ethical demand that medical and ethical decisions should be based on reliable and valid information, not on whatever information might have been gathered together arbitrarily or that might otherwise be susceptible to bias. Rather, for EBM and evidence-based ethics the particular ethical and methodological challenges are to analyze the normative judgments for distinguishing evidence from empirical information per se and account for them in practice with sufficient transparency. Before we can critically analyze this normativity implicit in the dimension of quality assessment of empirical information we need to have sufficient transparency about these value judgments in the first place.

### What an evidence-based ethics should be

**Figure 2:**
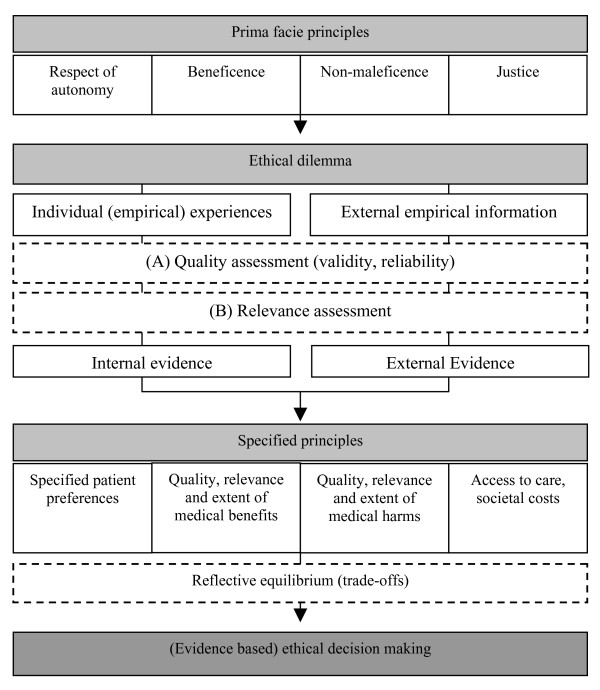
The process of decision-making in an evidence-based ethics.

In order to analyze the particularities of an evidence-based ethics, the following will begin by outlining a conception of the relation between normative and empirically descriptive statements. The relation between norms and facts has become an occasion of controversial debate in the past years, with the notable key terms including the "empirical turn in bioethics" [13] and the "social science critique of bioethics" [11]. This discussion cannot be recapitulated in any detail here. In a nutshell, as it relates to applied ethics, we could paraphrase Kant: thoughts (ethical principles, norms) without content (empirical information, evidence) are empty, intuitions (empirical information, evidence) without concepts (ethical principles, norms) are blind. [24] p. 130 (Translation by the author (DS). The parenthetical text is the author's addition). Thus normative and empirical statements should not be seen as competing for justificatory authority in ethical decisions, and hence there is not necessarily any danger of running into the naturalistic fallacy (see above). Rather, we should see the relation between norms and facts in the decision-making process as complementary. Each is necessary but not by itself sufficient for decision-making in applied ethics. The role of both ethical principles and empirical information is shown schematically in figure 2.

The classical principles of medical ethics [25] need to be given more specific content in actual cases – the principles of beneficence and non-harm, for example, only take on real concrete form once the dimensions of benefit and harm have a clear and specific content. To assess the justness of an action we need empirical data on distribution or on the possibilities of access to the health care system. Patient autonomy in turn is tied to the availability of patient preferences (individual or group-specific), which also have to be ascertained empirically.

Besides this interactional scheme, the figure also shows the characteristic normative dimensions of an evidence-based ethics. In the processes of quality assessment (A) and relevance assessment (B) typically there is some available external empirical information that does not reach the status of evidence. We could picture these processes as a sieve that sorts out some empirical information and leaves behind only *external evidence*. Only after a sufficiently critical assessment of the internal and external validity of the available empirical information are we justified in speaking of external evidence as the foundation for the specification of ethical principles. In principle we could conceive a similar process for one's own (empirical) experiences. In an ethical dilemma one could classify one's previous experiences as potentially distorted (quality assessment) or as not relevant for the case at hand (relevance assessment). Those experiences considered adequate and relevant could be termed internal evidence, in analogy to external evidence. However, since this assessment process cannot be examined intersubjectively and remains of necessity purely subjective, this internal evidence cannot be counted as evidence in the sense of evidence-based ethics (see the dimension of externality).

According to the interactional scheme in figure 2, a reflective and/or deliberative element has to precede the decision at the end in evidence-based ethics as well. The weighing of specified principles against each other is basically no different from what coherence theories of ethics call *reflective equilibrium *[25,26]. In reflective equilibrium the ethical and conceptual aspects have to be weighed against each other together with empirical information. This reflective equilibrium is susceptible to bias when methodologically bad and hence false or potentially misleading empirical information is taken into consideration. An evidence-based ethics only makes use of the best empirical information available in the case at hand in reflective equilibrium and sets (context-specific) minimal standards for the quality of empirical information that deserves the label of *external evidence*.

### The limits of an evidence-based ethics in practice

Having presented a rational and non-trivial reading of evidence-based ethics as a theoretical conception, this paper is now particularly concerned to identify the limits of evidence-based ethics in practice and the risks that go along with it. Whether the idealized conception of an evidence-based ethics presented in the previous section can be realized in the conditions of actual practice depends on several factors.

Before we can speak of evidence-based ethical decisions in practice, we have to demonstrate the extent to which we can assume a consensus on the specific use of the concept of evidence. To avoid misuse of the dimension of action legitimation (see above) or at least constrain it within certain limits we need a transparent justification for the use of the EB concept. This justification is not found in the articles cited at the beginning of this paper that use the concept of evidence-based ethics [2-5].

Another practical problem facing us is how to provide the EB concept with specific content depending on the methods used in studies. For example, the results of psychometric studies to determine the decision-making competence of patients with dementia could be relevant to research ethics. In the ethical discussion of advance health care directives, in turn, the results of quantitative and/or qualitative interview studies to determine patient preferences can be relevant. Various studies are available for both of these problem areas that can be expected to vary greatly in their validity and reliability [27,28]. Which of these studies should be considered evidence according to the assessment steps (A) and (B) in figure 2? We need objective criteria or adequate procedures for reaching consensus in order to justify why certain ethically relevant empirical information does or does not deserve the status of evidence. Of course this condition can only be realized if objective criteria are available to distinguish evidence from other ethically relevant empirical information. Here we still find great obstacles in practice, which again can be characterized by comparison to the practice of EBM. As already discussed, there is a contentious discussion within EBM as well concerning what one "may" or "should" with justification call "evidence-based". We can distinguish between two different problematic situations in EBM practice. Firstly, it is often a matter of contention whether certain medical decisions or recommendations are at all evidence-based or not, where some people consider the criteria for EBM to be satisfied and others not (Goodman). An example is the discussion of the medical benefit of Alzheimer's treatments. Several studies taking a general view of the situation conclude that their benefit is evidence-based [29,30], while others point to methodological flaws in the studies and take a very critical stance towards the use of the EB concept in this context [17,31]. A second example can be found in the discussion of the benefit of mammography screening for early detection of breast cancer. Again several studies speak in favor of an evidential basis of its benefit [32] while others come to the opposite conclusion and argue that to the contrary, the preponderance of harm over benefit is evidence-based [33]. The normative judgments mentioned above that go into the assessment of the quality of studies play a decisive role in these differences. This presents modern medicine as well as applied ethics with a practical problem of ethics and decision theory.

What problems does all this imply for an evidence-based ethics? As long as the relevant evidence of evidence-based ethics relates to the beneficial and harmful results of medical interventions, we can assume similar problems to those described in medicine. However, a more in-depth look at the practice of evidence-based ethics will have to consider the fact that most studies grouped under the heading "empirical ethics" use non-experimental methods taken from the social sciences. Quantitative and qualitative interview and questionnaire studies are conducted quite frequently in the course of projects on applied ethics [20,28]. These research methods can generate valuable empirical information for ethics. The goal of such investigations could be to determine patient preferences, the values of certain stakeholders in the field of health care, or attitudes and experiences with certain informed-consent procedures. Here as well studies can demonstrate better or worse methodological quality (internal validity, reliability) and can be more or less generalizable (external validity). Yet the discussion of when we are justified in calling the results of these types of studies evidence is still in its very beginning stages. The assessment of the internal and external validity of qualitative interview research in particular is the subject of much controversy [23,34]. This discussion also gives rise to the question of whether validity and reliability criteria as they have traditionally been used can even be applied to these types of studies [35]. But for quantitative research that uses questionnaires we also still lack a generally accepted gold standard of quality assessment [36,37].

These problems do not speak against carrying out these sorts of studies or using the results in making ethical decisions. They do, however, clearly point to the practical problems that arise in using the concept of evidence-based ethics. Without the appropriate tools to distinguish better and worse empirical studies, the EB concept cannot find any application in ethics.

## Conclusion

A rational, non-trivial reading of the EB concept has to be distinguished from empirical information per se. Because of its normative dimension of action legitimation, we need a transparent and rational justification of the context-specific use of the EB concept in medicine as well as in ethics. This is to be ensured through an explicit discussion within each field as to the validity and relevance of the empirical information to be considered evidence.

The relation between norms and facts was described as complementary in applied ethics. Empirical information per se is necessary to give concrete and context-specific reality to ethical principles. Yet neither empirical information nor ethical principles are sufficient for an ethical decision-making process in the context of medical ethics. The necessary interdependence of norms and facts is not sufficient to fully characterize the concept of evidence-based ethics. A rational, non-trivial reading of evidence-based ethics is characterized by a well-justified, context-specific differentiation between empirical information per se and the more qualitatively valuable evidence that has greater weight in the legitimation of action.

Yet so long as no criteria or standards with sufficient general acceptance are available to justify a transparent characterization of empirical information as evidence, we should refrain from using the EB concept in the context of applied ethics. An unexamined use of the EB concept in applied ethics without context-specific justification should be seen very critically due to its legitimating effect on actions. Hence collaborative, interdisciplinary work is needed, for example between professional societies in medical ethics and the social sciences, to work out agreed-upon criteria and standards. These standards for quality assessment in empirical ethics could then be used to assess research proposals or manuscripts submitted to journals. They could also be helpful in critically interpreting the results of studies in empirical ethics.

Until these quality assessment measures can be found for empirical ethics, it is likewise problematic to speak of "empirically supported ethical decisions" if there is no differentiation between various levels of quality of empirical information and hence no transparent discussion of the internal and external quality of the empirical information. A middle course between the evidence-based ethics that is not currently possible and a merely superficial treatment of empirical information is an ethics that calls for the *critical appraisal of empirical information in the context of totality of data*. Here, in a first step, systematic reviews aim to identify all studies that focused on research questions relevant for a certain ethical dilemma [38]. In a second step the review need to critically appraise, compare and discuss the empirical findings. The critical appraisal includes the following three aspects: (i) the validity of the data, (ii) the transferability of the data to the context under discussion and (iii) the relevance of the results for the decisions or recommendations at issue. In the discussion one has to interpret and qualitatively compare findings of different studies that investigated similar research questions.

A critical appraisal of empirical ethics can only be implemented in practice if the question of what comprises better or worse empirical information in ethics can be intersubjectively discussed and negotiated. This article has presented various difficulties that require further pragmatic discussion for their solution. Furthering this process of clarification, which has been neglected so far, is at least as important as the current intensive discussion about the relation between ethics and empirical information.

## Competing interests

The author declares that he has no competing interests.

## Pre-publication history

The pre-publication history for this paper can be accessed here:



## References

[B1] Sackett DL, Rosenberg WM, Gray JA, Haynes RB, Richardson WS (1996). Evidence based medicine: what it is and what it isn't. BMJ.

[B2] Major-Kincade TL, Tyson JE, Kennedy KA (2001). Training pediatric house staff in evidence-based ethics: an exploratory controlled trial. J Perinatol.

[B3] Tyson J (1995). Evidence-based ethics and the care of premature infants. Future Child.

[B4] Roberts LW (2000). Evidence-based ethics and informed consent in mental illness research. Arch Gen Psychiatry.

[B5] Jansen RP (1997). Evidence-based ethics and the regulation of reproduction. Hum Reprod.

[B6] Borry P, Schotsmans P, Dierickx K (2006). Evidence-based medicine and its role in ethical decision-making. J Eval Clin Pract.

[B7] Kim SY (2004). Evidence-based ethics for neurology and psychiatry research. NeuroRx.

[B8] Goldenberg MJ (2005). Evidence-based ethics? On evidence-based practice and the "empirical turn" from normative bioethics. BMC Med Ethics.

[B9] Loughlin M (2006). A platitude too far: 'Evidence-based ethics'. Commentary on Borry (2006), Evidence-based medicine and its role in ethical decision-making. Journal of Evaluation in Clinical Practice.

[B10] Sackett DL (1992). Evidence based medicine. JAMA.

[B11] Hedgecoe AM (2004). Critical Bioethics: Beyond the Social Science Critique of Applied Ethics. Bioethics.

[B12] Barbour RS (2001). Checklists for improving rigour in qualitative research: a case of the tail wagging the dog?. BMJ.

[B13] Sugarman J, Faden R, Weinstein J, Sugarman J, S D (2001). A Decade of Empirical Research in Medical Ethics. Methods in Medical Ethics.

[B14] Sackett DL, Straus S, Scott Richardson W (2000). Evidence-Based Medicine. How to Practice and Teach EBM.

[B15] Fletcher RW, Fletcher SW (2005). Clinical Epidemiology. The Essentials.

[B16] Goodman SN (2002). The Mammography Dilemma: A Crisis for Evidence-Based Medicine?. Ann Intern Med.

[B17] Pelosi AJ, McNulty SV, Jackson GA (2006). Role of cholinesterase inhibitors in dementia care needs rethinking. BMJ.

[B18] Strech D, Tilburt JC (2008). Value judgments in the analysis and synthesis of evidence. J Clin Epidemiol.

[B19] Richardson HS (2000). Specifying, balancing, and interpreting bioethical principles. J Med Philos.

[B20] Borry P, Schotsmans P, Dierickx K (2006). Empirical research in bioethical journals. A quantitative analysis. J Med Ethics.

[B21] Molewijk AC, Stiggelbout AM, Otten W, Dupuis HM, Kievit J (2003). Implicit Normativity in Evidence-Based Medicine: A Plea for Integrated Empirical Ethics Research. Health Care Analysis.

[B22] Altman DG, Schulz KF, Moher D, Egger M, Davidoff F, Elbourne D (2001). The revised CONSORT statement for reporting randomized trials: explanation and elaboration. The CONSORT Group. Ann Intern Med.

[B23] Malterud K (2001). Qualitative research: standards, challenges, and guidelines. Lancet.

[B24] Kant I (1992). Kritik der reinen Vernunft.

[B25] Beauchamp TL, Childress JF (2008). Principles of Biomedical Ethics.

[B26] Rawls J (1971). A theory of justice.

[B27] Kim SY, Karlawish JH, Caine ED (2002). Current state of research on decision-making competence of cognitively impaired elderly persons. Am J Geriatr Psychiatry.

[B28] Redman BK (2006). Review of measurement instruments in clinical and research ethics, 1999–2003. J Med Ethics.

[B29] Birks JS, Harvey R (2003). Donepezil for dementia due to Alzheimer's disease. Cochrane Database Syst Rev.

[B30] Olin J, Schneider L (2002). Galantamine for Alzheimer's disease. Cochrane Database Syst Rev.

[B31] Kaduszkiewicz H, Zimmermann T, Beck-Bornholdt HP, Bussche H van den (2005). Cholinesterase inhibitors for patients with Alzheimer's disease: systematic review of randomised clinical trials. BMJ.

[B32] Humphrey LL, Helfand M, Chan BK, Woolf SH (2002). Breast cancer screening: a summary of the evidence for the U.S. Preventive Services Task Force. Ann Intern Med.

[B33] Gotzsche PC, Olsen O (2000). Is screening for breast cancer with mammography justifiable?. Lancet.

[B34] Walsh D, Downe S (2006). Appraising the quality of qualitative research. Midwifery.

[B35] Giacomini MK, Cook DJ (2000). Users' guides to the medical literature: XXIII. Qualitative research in health care B. What are the results and how do they help me care for my patients? Evidence-Based Medicine Working Group. JAMA.

[B36] Dixon-Woods M, Agarwal S, Jones D, Young B, Sutton A (2005). Synthesising qualitative and quantitative evidence: a review of possible methods. J Health Serv Res Policy.

[B37] Pearlman RA, Starks HE, Sugarman J, Sulmasy DP (2001). Quantitative Surveys. Methods in Medical Ethics.

[B38] Strech D, Synofzik M, Marckmann G (2008). Systematic Reviews of Empirical Bioethics. Conceptual Challenges and Practical Recommendations. J Med Ethics.

